# Evaluation of the Effect of Repeated Intravitreal Bevacizumab Injections on the Macular Microvasculature of a Diabetic Patient Using Optical Coherence Tomography Angiography

**DOI:** 10.1155/2019/3936168

**Published:** 2019-04-18

**Authors:** Ayman G. Elnahry, Ahmed A. Abdel-Kader, Karim A. Raafat, Khaled Elrakhawy

**Affiliations:** Department of Ophthalmology, Faculty of Medicine, Cairo University, Cairo, Egypt

## Abstract

A 53-year-old female patient with center-involving diabetic macular edema affecting the left eye was imaged using optical coherence tomography angiography (OCTA) in both eyes. She underwent three monthly intravitreal bevacizumab injections in the left eye only and OCTA was repeated in both eyes one month following the last injection and showed decreased vascular density (VD) in the treated left eye but not in the untreated right eye compared to baseline. No further injections were required in either eye, and OCTA was done in both eyes 4 months following the last injection which showed improved VD of the left eye with stable VD in the right. Three monthly intravitreal bevacizumab injections were then required in both eyes; then OCTA was repeated following the last injection and revealed decreased VD in both eyes compared to previous scan. OCTA could be a useful tool for detecting VD changes following bevacizumab injections in diabetics.

## 1. Introduction

Diabetic macular edema (DME) is a major cause of vision loss worldwide that is partly mediated by excess vascular endothelial growth factor (VEGF) production by hypoxic and damaged retinal tissues. Several agents, including bevacizumab, aimed at targeting and blocking VEGF molecules, have been developed, and have proven useful in the treatment of DME [1, 2]. Because the action of these agents, however, is not long lasting, treatment may be required for extended periods which raises the concern for long term effects of these agents such as worsening of macular perfusion through increasing capillary nonperfusion and vasoconstriction [2, 3].

Studies evaluating macular perfusion following intravitreal anti-VEGF injections have mainly relied on fluorescein angiography (FA) [4]. Optical coherence tomography angiography (OCTA) is a new noninvasive modality capable of imaging the different retinal capillary layers of the macula in greater detail compared to conventional FA. Unlike FA, it does not need dye injection and uses several technologies, including the split-spectrum amplitude decorrelation angiography (SSADA) algorithm, to image blood flow in the retinal and choroidal blood vessels [5]. Previously, we and others have shown that OCTA can detect areas of capillary nonperfusion not appreciated on fluorescein angiography [6, 7].

We evaluated the effect of repeated intravitreal bevacizumab injections on the capillary density of the maculae of a diabetic patient using OCTA.

## 2. Case Report

**Figure 1:**
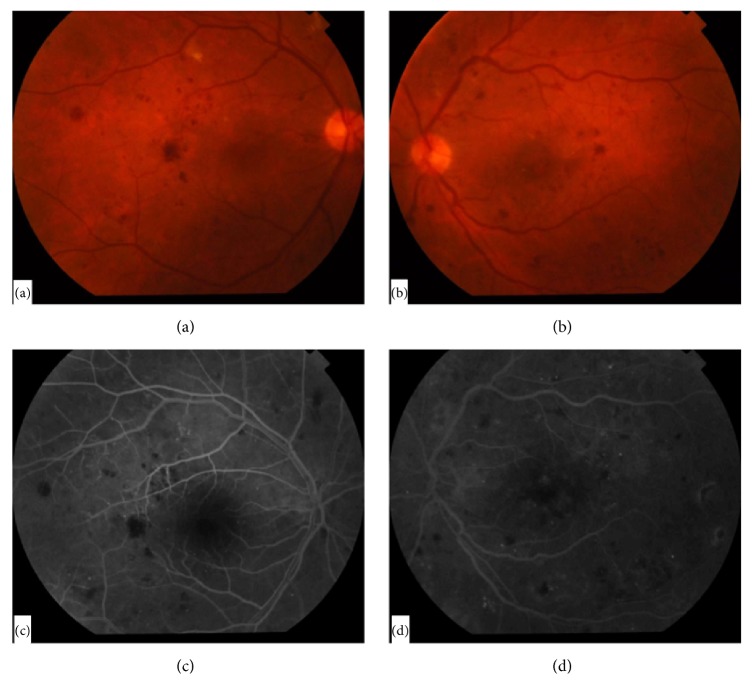
Fundus photography and fluorescein angiography of both eyes. Fundus photography showed retinal hemorrhages affecting the posterior pole in both eyes with clinically significant macular edema in the left eye (a and b). Fluorescein angiography revealed microaneurysms and areas of blocked fluorescence due to retinal hemorrhages in both eyes with leakage of fluorescein in the left macula (c and d).

**Figure 2:**
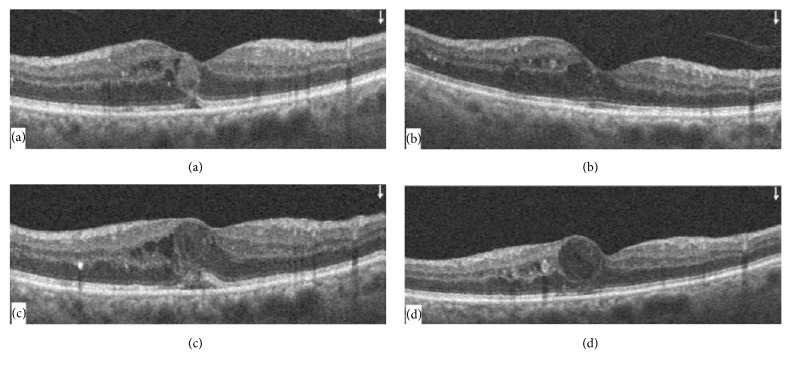
Structural OCT of the left macula. (a) At presentation, there were multiple intraretinal cystic spaces with mild subfoveal neurosensory detachment and diffuse retinal thickening. (b) Following 3 monthly intravitreal bevacizumab injections, there was improvement of the retinal edema and neurosensory detachment. (c) Follow-up 4 months following the last injection showed increased intraretinal cystic spaces and retinal thickness with recurrence of neurosensory detachment. (d) Following another 3 intravitreal injections, there was improvement of the neurosensory detachment and retinal thickness.

**Figure 3:**
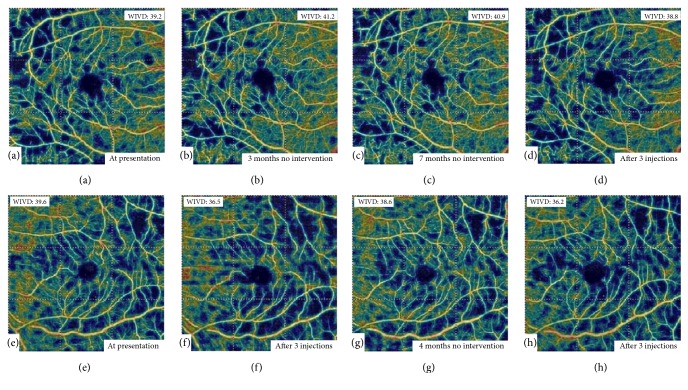
OCTA of the SCP of the right (top row) and left (bottom row) maculae. At presentation, there were areas of capillary nonperfusion detected in the SCP of both eyes especially temporally (a and e). Following three months of no intervention in the right eye and three monthly intravitreal injections in the left eye, there was increased vascular density (VD) in the right eye (b) and decreased VD in the left (f). After four months of no interventions in either eye, there was no change in the VD in the right eye (c) and increased VD in the left (g). Finally, following three monthly intravitreal injections in both eyes, there was decreased VD in both eyes (d and h). Scan quality was 6/10 or higher in all images and there were no significant segmentation errors detected. Whole image vascular density (WIVD), as measured by the built-in machine software, is shown at each visit.

A 53-year-old female presented with gradually progressive diminution of vision in her left eye for 2 months. She had a history of diabetes mellitus for 20 years for which she is taking insulin. Her most recent HbA1C was 7.5%. She had no history of hypertension or renal problems. She had no past ocular history. Examination revealed a corrected distance visual acuity (CDVA) of 20/40 in her right eye and 20/100 in the left. Anterior segment examination showed nuclear sclerosis in both eyes. Posterior segment examination revealed intraretinal hemorrhages in all 4 quadrants indicating severe nonproliferative diabetic retinopathy in both eyes with clinically significant macular edema in the left eye confirmed by fluorescein angiography (Figure 1). Spectral domain optical coherence tomography (OCT) of the macula was done and revealed multiple cystic spaces, mild subfoveal neurosensory detachment, and diffuse retinal thickening with a central subfield macular thickness of 332 *μ*m in the left eye (Figure 2(a)). The right eye showed only few cystic spaces with minimal thickening. OCTA (Optovue, Inc., Fremont, CA, USA) was done in both eyes and showed areas of capillary nonperfusion in the superficial capillary plexus (SCP) of the maculae of both eyes (Figures 3(a) and 3(e)). Three monthly intravitreal bevacizumab injections were done to treat the macular edema in the left eye. One month following the last intravitreal injection, CDVA improved to 20/60 in the left eye and was stable in the right eye. OCT showed improvement of the macular edema in the left eye (Figure 2(b)). OCTA was performed in both eyes and showed decreased vascular density of the SCP of the left eye compared to pretreatment OCTA while a mild increase was noted in the vascular density of the SCP of the untreated right eye (Figures 3(b) and 3(f)). The patient was then followed up without requiring further intravitreal injections and 4 months following the last intravitreal injection OCTA was repeated in both eyes and showed improvement of the vascular density of the SCP of the left eye with unchanged SCP in the right eye (Figures 3(c) and 3(g)). CDVA was 20/60 in the right eye and 20/100 in the left. Clinical examination revealed retinal neovascularization in the right eye with clinically significant macular edema in both eyes. OCT showed increased center-involving macular edema in both eyes with recurrent neurosensory detachment in the left eye (Figure 2(c)). Three monthly intravitreal bevacizumab injections were then done for treatment of proliferative diabetic retinopathy in the right eye and the macular edema in both eyes. One month following the last intravitreal injection, CDVA was 20/40 in the right eye and 20/60 in the left, OCT showed decreased macular thickness in both eyes (Figure 2(d)), and OCTA revealed decreased vascular density of the SCP of both eyes (Figures 3(d) and 3(h)). Changes in the deep capillary plexus closely followed those in the SCP at all stages.

## 3. Discussion

According to the World Health Organization criteria on drugs and adverse drug reactions, a certain association is present between the drug and the adverse event, including an abnormal laboratory test, if the event occurs in a plausible time relationship to drug administration and could not be explained by any other factor. The response to withdrawal of the drug, known as dechallenge, should be plausible, and the event must be definitive pharmacologically and using a satisfactory rechallenge procedure if necessary [8]. These criteria were all fulfilled in the presented case.

In a previous study assessing the effect of bevacizumab injections on the macular perfusion in diabetic patients without severe macular ischemia, there was no worsening of macular ischemia following 3 intravitreal injections given at 6 weeks' interval. This study, however, relied on FA for this assessment and assessed mainly parameters related to the foveal avascular zone which may not be fully representative of the whole macular perfusion [2, 4]. In another study evaluating the visual outcomes of bevacizumab injections in patients with DME with and without macular ischemia defined by FA, there was a negative effect from macular ischemia on the visual outcome of injections [9]. This may indicate further worsening of macular perfusion by VEGF inhibition in these cases [3, 9]. OCTA has been shown to be more sensitive in detecting areas of capillary nonperfusion when compared to FA and could therefore be more suited for assessing the status of the macular perfusion before and after anti-VEGF injections in diabetic patients [6, 7]. Because areas of capillary nonperfusion are more prevalent in diabetic patients in the SCP than the deep plexus as imaged by OCTA, with less projection artifacts, we focused our analysis on the SCP [7].

The increased areas of capillary nonperfusion detected by OCTA following bevacizumab injections could also be the result of slowing of blood flow in these capillaries rather than due to complete capillary obliteration. This would explain the apparent perfusion of these capillaries when imaged by FA but not OCTA, a phenomenon often observed with some microaneurysms detected by FA but not OCTA and suggested to be due to inability of the SSADA algorithm to detect retinal capillary flow less than 0.3 mm per second [7]. Indeed, intravitreal bevacizumab injections have been shown to reduce the mean blood flow velocities in the central retinal, posterior ciliary, and ophthalmic arteries of patients with wet age-related macular degeneration 4 weeks following a single intravitreal injection [10], an effect that may be more exaggerated in diabetic patients.

Lastly, there is a remote possibility that the increase and decrease in the capillary density observed with OCTA in this case may be a part of the natural history of diabetic retinopathy. This, however, is unlikely given that the capillary density decreased only when intravitreal bevacizumab injections were given but otherwise improved or was stable in the absence of injections in either eye.

This case, therefore, highlights the urgent need for further studies to evaluate the effect of repeated intravitreal bevacizumab injections, and other anti-VEGF agents, on the macular microvasculature of diabetic patients using the new flow-detecting OCTA imaging modality.
